# Intermittent Theta Burst Stimulation for Major Depressive Disorder with Comorbid Anxiety: A Systematic Review of Clinical Efficacy and Predictors of Response

**DOI:** 10.3390/brainsci16020167

**Published:** 2026-01-30

**Authors:** Deborah Maria Trandafir, Cristina Dumitru, Florin Zamfirache, Gabriela Narcisa Prundaru, Constantin Alexandru Ciobanu, Beatrice Mihaela Radu, Adela Magdalena Ciobanu

**Affiliations:** 1 Neuroscience Department, Discipline of Psychiatry, Faculty of Medicine, “Carol Davila” University of Medicine and Pharmacy, 020021 Bucharest, Romania; deborah-maria@drd.umfcd.ro (D.M.T.); adela.ciobanu@umfcd.ro (A.M.C.); 2 Department of Psychiatry, “Prof. Dr. Alexandru Obregia” Clinical Hospital of Psychiatry, 041914 Bucharest, Romania; 3 Department of Anatomy, Animal Physiology and Biophysics, Faculty of Biology, University of Bucharest, Splaiul Independenţei 91-95, 050095 Bucharest, Romania; zamfirache.florin@s.bio.unibuc.ro (F.Z.); prundaru.gabriela-narcisa@s.bio.unibuc.ro (G.N.P.); 4 Department of Educational Sciences, Faculty of Educational Sciences, Social Sciences and Psychology, The National University of Science and Technology POLITEHNICA Bucharest, Pitești University Centre, Targul din Vale, 1, 110040 Pitesti, Romania; cristina.dumitru81@upb.ro; 5 Faculty of Medicine, “Carol Davila” University of Medicine and Pharmacy, 020022 Bucharest, Romania; alexandru.ciobanu2020@stud.umfcd.ro

**Keywords:** anxiety disorders, comorbidity, clinical efficacy, intermittent theta-burst stimulation, repetitive transcranial magnetic stimulation, major depressive disorder, neuromodulation

## Abstract

**Background:** Intermittent theta burst stimulation (iTBS), a patterned form of repetitive transcranial magnetic stimulation (rTMS), has gained increasing attention as a time-efficient neuromodulation protocol for major depressive disorder (MDD). However, its clinical effectiveness in individuals with co-occurring depression and anxiety remains insufficiently characterized. This systematic review aimed to evaluate clinical outcomes, including depressive and anxiety symptom severity, response, and remission, following rTMS in individuals with major depressive disorder and elevated anxiety symptoms. The primary outcome was the reduction of depressive and anxiety symptoms, while secondary outcomes included response and remission rates, adverse events, and potential predictors of treatment response. **Methods:** A systematic search was performed following the PRISMA guidelines in the following databases: PubMed, Scopus, Embase, PsycInfo, Web of Science, Elsevier, Google Scholar. The protocol was registered in PROSPERO (CRD420251117784). Six studies that met the inclusion criteria were selected as eligible; these included one randomized controlled trial, one controlled clinical trial, three open-label studies, and one retrospective study on iTBS alone or compared to conventional 10 Hz rTMS or pharmacotherapy. iTBS has demonstrated safety and efficacy in reducing depressive and anxiety symptoms. The response rate ranged between 30 and 60%, and the remission rate between 10 and 40%. Regarding comparative findings, the results are mixed, with some studies showing superior or comparable improvements to 10 Hz rTMS and others reporting no significant differences. Reported treatment outcomes were largely influenced by age, baseline severity, medication status, and comorbid anxiety. Antipsychotics, anticonvulsants, and benzodiazepines were associated with attenuated clinical benefit, and bupropion use was associated with increased response. **Conclusions:** Current evidence supports iTBS as an effective, well-tolerated, and time-efficient intervention for adults with depression and comorbid anxiety. However, variability in treatment outcomes and limited mechanistic data highlight the need for larger, harmonized, and mechanistically informed randomized trials to refine stimulation parameters, improve patient stratification, and clarify the neurobiological substrates of treatment response.

## 1. Introduction

Major depressive disorder (MDD) and anxiety disorders are the most common and disabling psychiatric conditions worldwide, often occurring together and generating a complex and variable form of clinical presentation. Although historically treated as separate diagnostic categories, contemporary epidemiological and neurobiological research highlights significant overlap between them [1,2]. Estimates indicate that between 45% and 67% of individuals with MDD also meet criteria for at least one anxiety disorder, while 30–63% of those with anxiety disorders simultaneously experience depressive symptoms. This comorbidity leads to greater severity of the illness, decreased treatment response rate, and increased risk of relapse [3]. Anxious depression emerges in longitudinal analyses as a chronic and treatment-resistant profile, with greater persistence of symptoms compared to non-anxious major depression [3].

Depression and anxiety present partially overlapping but distinct patterns of neural dysfunction from a neurobiological perspective. Both involve alterations in cortico-limbic networks (amygdala, hippocampus, and anterior cingulate cortex), regions involved in emotion regulation and threat processing. MDD is typically described by hypoactivation of the dorsolateral prefrontal cortex (DLPFC) and hyperactivation of limbic structures, such as the amygdala [4]. In the case of anxious depression, these differences are more pronounced, with increased reactivity and lower conflict monitoring [2]. Recent studies on functional connectivity have demonstrated disruptions in theta band activity of fronto-limbic circuits in people with anxious depression, highlighting a disruption of neural rhythms involved in attention control, affective regulation, and synaptic plasticity [5,6,7]. Also, in MDD, functional interaction between DMN and subgenual prefrontal cortex leads to depressive rumination [8].

These combined results increase interest in neuromodulatory interventions aimed at restoring cortical excitability and rebalancing dysfunctional neural circuits. Repetitive transcranial magnetic stimulation (rTMS) is a well-established therapeutic approach for depression, but conventional high-frequency stimulation yields modest results, with response and remission rates remaining relatively limited [9]. Theta burst stimulation (TBS), developed to mimic the specific patterns of theta frequency activation that are often associated with learning and plasticity, has been identified as a more physiologically effective alternative. Intermittent TBS (iTBS) produces excitatory effects comparable to 10 Hz rTMS, but has a significantly shorter protocol duration [10]. Accordingly, evidence from a large randomized controlled trial indicates that iTBS is non-inferior to 10 Hz rTMS with respect to symptom reduction, response, and remission rates, and shows comparable tolerability and safety in treatment-resistant depression [11]. Similarly, another randomized controlled trial showed that response and remission rates at one month were maintained at six months for both iTBS and 10 Hz rTMS [12]. This offers practical advantages for patients who experience difficulty adhering to treatment when treatment sessions are longer.

TBS protocols for anxiety-related symptomatology are highlighted by a growing body of clinical and neuroimaging evidence. Studies of iTBS in DLPFC have reported reductions in generalized anxiety, autonomic arousal, and worry severity, along with increased prefrontal activation and reduced hyperconnectivity in limbic networks [13,14,15]. Research examining the effects of iTBS compared to continuous TBS (cTBS) suggests that the choice of excitatory versus inhibitory protocols may influence treatment outcomes, with iTBS often associated with stronger antidepressant effects, while cTBS appears to modulate anxiety-related hyperactivation and suicidality in certain subgroups of patients [16].

The clinical efficacy of iTBS in anxious depression, despite all the studies conducted, still remains inconsistent. Studies differ significantly in terms of parameters, duration, location and concomitant pharmacological treatment. At the same time, anxiety symptoms may influence the reactivity of neuromodulation through changes in the dopaminergic and serotonergic systems [14,17].

This systematic review aims to consolidate the available evidence on the clinical efficacy, tolerability, and neurobiological impact of iTBS in adults with MDD with comorbid anxiety symptoms or disorders. This review assesses whether iTBS offers advantages over traditional rTMS paradigms and identifies factors that may influence treatment response in patients with overlapping depression and anxiety.

## 2. Review Objectives and Research Questions

Although the antidepressant efficacy of iTBS is increasingly being investigated, evidence regarding its efficacy in patients with comorbid depression and anxiety remains limited. This systematic review was designed to clarify the therapeutic role of iTBS.

This review evaluates the effectiveness of iTBS in reducing depressive and anxiety symptoms in adults diagnosed with MDD, anxiety disorders, or comorbid conditions.

Second, it examines reported response rates and remission rates to determine the consistency of clinical improvement across studies. Third, it identifies methodological and clinical factors associated with variability in treatment outcomes, including stimulation parameters, cortical targets, baseline symptom severity, and concomitant pharmacotherapy. It evaluates comparative analyses to determine whether treatment effects differ between iTBS administered as a stand-alone intervention and iTBS used in combination with other rTMS modalities or adjunctive therapies.

This review applies a structured, PRISMA-guided approach to identify, review, and synthesize data from included randomized, controlled clinical trials, open-label studies, and retrospective studies. This methodology follows an assessment of current studies and aims to identify gaps that require further investigation to optimize neuromodulation strategies for mood and anxiety comorbidity.

The analysis addressed the following research questions:

Q1: What is the effectiveness of iTBS in reducing depressive and anxiety symptoms in adults with comorbid MDD and anxiety disorders?

Q2: What response and remission rates have been reported for iTBS in these populations?

Q3: What factors (e.g., details of the stimulation protocol, target site, baseline severity, and concomitant medication use) are associated with variability in treatment outcomes?

## 3. Materials and Methods

### 3.1. Study Protocol

This systematic review was conducted in accordance with the Preferred Reporting Items for Systematic Reviews and Meta-Analyses (PRISMA) guidelines [18,19]. The review’s primary objectives and methodological framework were prospectively registered in the PROSPERO database under the identifier CRD420251117784. Consistent with the PROSPERO-registered protocol, mechanistic evidence was included only descriptively when reported within eligible clinical studies; no separate search or quantitative synthesis of mechanistic data was conducted.

### 3.2. Identification of Studies

The search aimed to identify experimental and clinical studies evaluating the effects of iTBS on depressive and anxiety outcomes in adults diagnosed with MDD with comorbid anxiety symptoms or disorders. A systematic literature search was conducted in accordance with PRISMA guidelines across the following electronic databases: PubMed/MEDLINE, Embase, PsycInfo, Web of Science, Scopus, Elsevier, and Google Scholar. Searches covered all records from database inception to 5 August 2025. Google Scholar was included to capture grey literature and additional peer-reviewed studies not always indexed consistently in traditional biomedical databases; for feasibility and to ensure relevance, screening was restricted to the first 200 search results, which Google Scholar ranks by citation metrics and query relevance, a strategy commonly used in systematic reviews to balance comprehensiveness with methodological rigour. Database-specific search strategies were developed to maximize sensitivity while maintaining relevance. Controlled vocabulary terms (MeSH in PubMed; Emtree in Embase) were used where applicable and combined with free-text terms. Searches were performed primarily in the title and abstract fields, with database-specific keyword fields included when available. The core search concepts included: (1) intermittent theta burst stimulation, (2) depressive disorders, and (3) anxiety disorders. The search strategy used the following Boolean string applied to titles and abstracts: (“intermittent theta burst stimulation” OR “iTBS”) AND (“depression” OR “major depressive disorder”) AND (“anxiety” OR “anxiety disorders”). For PubMed, the search strategy was as follows (“intermittent theta burst stimulation” OR iTBS OR “theta burst stimulation” [MeSH]) AND (“major depressive disorder” OR depression [MeSH] OR depressive disorder) AND (anxiety OR “anxiety disorders” [MeSH]). Filters were applied to restrict results to English-language, human, adult, and full-text studies. Equivalent Boolean strategies were adapted for each database using appropriate controlled vocabulary and syntax (full search strings for each database are provided in Supplementary Table S1). Filters were applied to restrict results to human studies, adults (≥18 years), English-language publications, and full-text availability.

All retrieved records were imported into EndNote 2025 for reference management and duplicate removal. Title–abstract screening and full-text evaluation were performed independently by two reviewers to ensure methodological rigor and minimize selection bias. This approach enabled the comprehensive identification of empirical studies investigating iTBS and related rTMS protocols in adult populations with depression, anxiety, or their comorbidity.

The requirement that studies include both depression- and anxiety-related terms was intentionally applied to identify trials explicitly addressing comorbidity or prospectively assessing anxiety outcomes. As a consequence, depression-focused iTBS trials that measured anxiety only as an unindexed secondary outcome may not have been captured. This strategy was chosen to preserve conceptual specificity but may have reduced the overall yield.

### 3.3. Study Inclusion and Exclusion Criteria

To ensure methodological rigor and alignment with the research goals, predefined inclusion and exclusion criteria were used to select studies evaluating the efficacy of iTBS in adults with MDD, anxiety disorders, or comorbid conditions. For the purposes of this review, comorbid anxiety was defined as the presence of clinically significant anxiety symptoms in individuals with MDD, as assessed using validated anxiety rating scales (e.g., HAM-A, BAI, HADS-A), regardless of whether a formal DSM- or ICD-based anxiety disorder diagnosis was reported. Studies that explicitly reported diagnosed comorbid anxiety disorders were included where available; however, the majority of the evidence base reflects MDD populations characterized by elevated anxiety symptom severity rather than categorical comorbid anxiety diagnoses. Studies were eligible if they included adults with MDD in whom anxiety was assessed using validated symptom scales and exceeded clinically relevant thresholds. Formal DSM- or ICD-based diagnoses of anxiety disorders were not required for inclusion. Only studies meeting the criteria summarized in Table 1 were included.

Studies not fulfilling these requirements were excluded based on the criteria listed in Table 2, ensuring the methodological consistency and clinical relevance of the final dataset.

Although the review prioritized randomized controlled trials, three open-label studies and one retrospective study, without a sham comparator, were included due to their relevance to the clinical question and the limited availability of controlled trials in this population.

The review focused primarily on studies applying iTBS to DLPFC, the most common therapeutic target in depression treatment. Eligible comparisons included: (i) conventional 10 Hz rTMS, (ii) pharmacotherapy (e.g., sertraline).

Outcomes of interest primarily included validated measures of depressive and anxiety symptom severity, response, and remission. Mechanistic outcomes (e.g., neuroimaging or biological markers) were extracted when available but were not a primary inclusion criterion.

### 3.4. Study Selection

**Figure 1:**
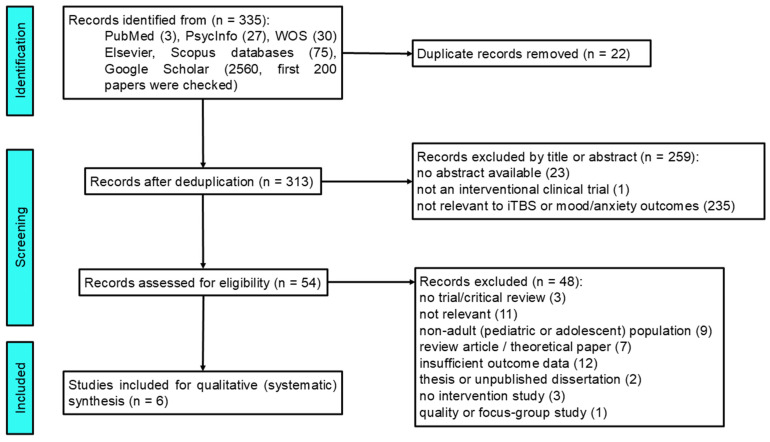
PRISMA flow diagram illustrating the study identification, screening, eligibility assessment, and inclusion process.

All retrieved articles were imported into EndNote (Clarivate Analytics) for reference management and duplicate removal. After removal of duplicate articles, the remaining records were analyzed in a two-stage process, in accordance with the PRISMA guidelines [18,19]. In the identification phase, database searches yielded a total of 335 records. Although Google Scholar returned several thousand results, only the first 200 records ranked by relevance were screened, consistent with established methodological practice. During the title and abstract screening stage, records were excluded if they were clearly irrelevant, non-interventional, review articles, or did not involve adult human participants. Full-text articles were then assessed for eligibility using predefined inclusion and exclusion criteria. Reasons for exclusion at this stage included non-adult populations, absence of iTBS/TBS intervention, lack of relevant outcome data, or inappropriate study design. Ultimately, six studies met all inclusion criteria and were included in the qualitative systematic synthesis. No quantitative meta-analysis was performed due to heterogeneity in study design, populations, and outcome measures. The full study selection process is illustrated in the revised PRISMA flow diagram (Figure 1).

In the first stage, two reviewers assessed titles and abstracts to eliminate clearly irrelevant studies, including animal research, pediatric samples, case reports, reviews, and non-interventional papers. In the second stage, full-text articles deemed potentially eligible were independently evaluated to determine whether they met all predefined inclusion criteria related to study design, participant characteristics, diagnostic standards, intervention type, and outcome measures. Where the reviewers disagreed, the situation was resolved through discussion and clarifications, with input from a third reviewer when required. The research selection process, including the number of records identified, screened, excluded (with reasons), and retained for analysis, is depicted in the PRISMA flow diagram (Figure 1).

### 3.5. Data Extraction and Quality Assessment

Relevant data collection was performed independently by the reviewers using a standardized data gathering format. Extracted data included: study characteristics (author, year, country, design, sample size), participant demographics and diagnostic details, intervention parameters (stimulation site, intensity, number and frequency of sessions, duration), control conditions (e.g., pharmacotherapy, 10 Hz rTMS), and primary and secondary outcomes (depressive and anxiety symptom changes, response and remission rates, adverse events).

When multiple reports originated from the same study cohort, the most complete or most recent dataset was retained. Any discrepancies between reviewers were resolved through discussion, with a third reviewer consulted when necessary.

Methodological quality and risk of bias were assessed using the Cochrane Risk of Bias 2 (RoB 2) tool [20], evaluating domains related to randomization, deviations from intended interventions, missing outcome data, outcome measurement, and selection of the reported result. Open-label and retrospective studies were evaluated using the ROBINS-I tool. Each study was rated as having low risk, some concerns, or high risk of bias.

The robustness of evidence for each key outcome (depression severity, anxiety severity, response/remission, and adverse events) was evaluated using the GRADE framework. Study-derived evidence from randomized trials was initially rated as high certainty and downgraded based on risk of bias, inconsistency, indirectness, imprecision, and publication bias. The risks of bias, inconsistency, indirectness, imprecision, as well as the overall certainty of evidence (GRADE), are summarized in Table 3.

According to the GRADE framework, the certainty of evidence across all efficacy outcomes was rated as very low. This was driven by serious risk of bias, substantial heterogeneity in stimulation parameters and study designs, imprecision related to small sample sizes, and indirectness resulting from inconsistent definitions of anxiety (symptom severity versus formal comorbid diagnosis). Certainty of evidence for adverse events was rated as low, reflecting consistent reporting of mild and transient side effects across studies.

A quantitative meta-analysis was not conducted due to substantial heterogeneity in stimulation parameters, study designs, outcome measures, and reporting of effect sizes, which precluded meaningful statistical pooling.

## 4. Results

### 4.1. Study Identification and Selection

Six studies were identified evaluating the effects of iTBS in adults with MDD, anxiety disorders, or comorbid conditions. As shown in Figure 1, the search process initially yielded 335 records, of which 313 remained after duplicate removal. Following title and abstract screening, 54 full-text articles were assessed for eligibility, and six studies met all inclusion criteria and were included in the final synthesis.

The included trials, published between 2000 and 2023, comprised 897 adult participants aged 18–65 years. Across studies, iTBS was predominantly applied to DLPFC, either as a standalone intervention or in combination with additional neuromodulation strategies (e.g., 1 Hz rTMS, HF-rTMS) or pharmacotherapy. Study designs varied, including one RCT, one controlled clinical trial, several open-label interventions, and one retrospective study.

Table 4 summarizes the primary characteristics of the included studies, including protocol parameters, stimulation intensity, target regions, sample characteristics, and reported clinical outcomes.

### 4.2. Methodological Quality and Risk of Bias Assessment

The methodological approach of the presented studies was evaluated to identify any potential bias and determine the reliability of the available evidence. Assessment followed the Cochrane Risk of Bias 2 (RoB 2) tool for RCTs, which evaluates bias across five domains: (1) randomization process, (2) deviations from intended interventions, (3) missing outcome data, (4) measurement of the outcome, and (5) selection of the reported result. Domain-level judgments (low risk, some concerns, or high risk) were made independently and are reported with brief justifications. Three open-label designs and one retrospective study did not include an active comparator. Given that RoB 2 is not intended for nonrandomized studies, these studies were not formally assessed using RoB 2; instead, their methodological limitations are described narratively, and their findings are interpreted with caution.

Of the included studies, one implemented appropriate randomization procedures and used validated clinical scales—including the Hamilton Depression Rating Scale (HAM-D), the Hamilton Anxiety Rating Scale (HAM-A)—to ensure a standardized outcome measure. Reported blinding procedures were inconsistent, particularly in studies involving pharmacological comparators or combined neuromodulation protocols, resulting in an increased risk of performance bias.

Reporting errors and participant attrition were low in all studies. Several studies did not include pre-registration or detailed descriptions of allocation procedures. Sample size limitations were common (*n* < 50 in many studies), thus limiting statistical power and generalizability. Heterogeneity in stimulation parameters, such as the number of pulses, stimulation intensity, session frequency, and variation in treatment combinations, contributed to additional methodological variability.

The included randomized trial was judged to have low risk or some concerns across RoB 2 domains. The most frequent sources of potential bias related to deviations from intended interventions and outcome measurement are particularly in trials with incomplete blinding. A summary of domain-level risk-of-bias judgments and justifications for the randomized and controlled studies is provided in Table 5. These results indicate that future research would benefit from larger, pre-registered RCTs that use standardized iTBS protocols and rigorous blinding methods to increase the robustness of causal interpretations.

The randomized study implemented adequate randomization procedures and exhibited low levels of attrition and reporting bias. One controlled study presented some concerns regarding the risk of bias. However, blinding practices were inconsistent, particularly in open-label designs and trials involving combined therapeutic approaches. The large-scale RCT [23] contributed the strongest methodological evidence, whereas smaller studies provided preliminary but informative findings supporting the potential efficacy of iTBS in populations with comorbid depression and anxiety. Risk of bias in the included open-label and retrospective studies was systematically assessed using the ROBINS-I tool, with the results summarized in Table 6.

Overall, the included studies were open-label and lacked comparator groups, introducing potential variability due to the natural course of illness, concomitant treatments, or placebo effects. The protocol for selecting participants either lacked randomization, clear procedures [13], or was based on standard clinical practice [21], making it difficult to generalize the results. Two studies described the iTBS intervention in detail, but add-on treatments varied considerably with concomitant antidepressants, psychotherapy, or were not mentioned at all. One open-label study [15] clearly explained the protocol and the parameters. There was a moderate risk of bias due to deviations from intended interventions. Follow-up and treatment adherence were not strictly controlled, and the open- label structure or placebo effects may have influenced the outcomes. Regarding the data, not all dropouts or missing data were fully reported, and the small sample size may have influenced the study findings. The risk of bias in outcome measurement was moderate due to the lack of blinding, and subjective measures could have influenced the consistency and objectivity of the assessments. Reporting bias across studies may have been present, given that many outcomes were reported mainly in terms of significant pre/post differences, whereas other results may have received less attention. In the retrospective study, the risk of bias is primarily due to the absence of a control group, lack of blinding, and exclusion of patients with incomplete clinical charts.

### 4.3. Characteristics of Included Studies and Intervention Protocols

All included studies examined the effects of iTBS in adults diagnosed with MDD, anxiety disorders, or comorbid presentations, with diagnoses established according to DSM-IV/5 or ICD-10 criteria. The included studies comprised one randomized controlled trial, one controlled clinical trial, three open-label studies, and one retrospective study. Sample sizes varied substantially, ranging from 37 to 414 participants, with the majority of individuals aged 18–65 years. Both male and female participants were represented, and none of the studies reported sex-specific differences in treatment outcomes.

Interventions predominantly involved iTBS applied to DLPFC, delivered either as a standalone protocol or combined with additional treatments, such as low-frequency right DLPFC rTMS, pharmacotherapy (e.g., sertraline or antidepressants), or psychotherapy. The included trials assessed the efficacy of iTBS relative to standard high-frequency (10 Hz) rTMS or pharmacotherapy. The duration of treatment in the selected studies ranged from 2 to 6 weeks, with an average frequency of 5 sessions per week and stimulation intensity between 80 and 120% of resting motor threshold. A number of impulses between 600 and 1600 were used.

Outcomes were measured using the Hamilton Depression Rating Scale (HAM-D/HDRS), the Hamilton Anxiety Rating Scale (HAM-A), the Patient Health Questionnaire (PHQ-9), the Generalized Anxiety Disorder Scale (GAD-7) and the Hospital Anxiety and Depression Scale (HADS), the Beck Depression Inventory (BDI), the Beck Anxiety Inventory (BAI), the Symptom Checklist-90 (SDS) and the Short Anxiety Symptom Inventory Subscale (BSI-A).

The most common outcomes reported were reductions in the severity of depressive and anxiety symptoms, improvements in emotional regulation, and overall clinical functioning. However, there was great diversity in stimulation parameters, treatment combinations, and outcome assessment. This variation likely explains the differences in efficacy reported across studies (see Table 7).

### 4.4. Participant Demographics and Clinical Baseline Features

A total of 897 adult participants (aged 18–65 years) were enrolled in the six studies. The majority of participants were diagnosed with MDD, either first-episode or recurrent. Several studies also included people with comorbid anxiety disorders, such as generalized anxiety disorder or mixed anxiety-depressive disorder. Diagnosis was made using DSM-IV, DSM-5, or ICD-10 criteria.

The mean age of participants was 35–40 years, with both male and female study samples. Clinical severity was generally moderate to severe, and the Hamilton Rating Scale for Depression (HAM-D), Hamilton Anxiety Rating Scale (HAM-A), Hospital Anxiety and Depression Scale (HADS), Patient Health Questionnaire (PHQ-9), and Generalized Anxiety Disorder Scale (GAD-7) were used to assess this.

Most participants were outpatients receiving concomitant pharmacological treatment, most often selective serotonin reuptake inhibitors (SSRIs) such as sertraline or other antidepressants such as bupropion.

Exclusion criteria included neurological disorders, current substance abuse, recent electroconvulsive therapy, or contraindications to transcranial magnetic stimulation (TMS). Psychotherapy or supportive counseling, in routine clinical practice, was reported in several studies. Overall, participant characteristics were largely similar across studies, supporting the correct interpretation of the pooled results regarding the efficacy of iTBS for symptoms of depression and anxiety in people with anxiety-related comorbidity. Across heterogeneous studies, rTMS was associated with symptom improvement, particularly for depressive outcomes, although findings remain inconsistent and largely qualitative.

### 4.5. Stimulation Parameters and Treatment Conditions Across Trials

In all studies, iTBS was consistently applied to DLPFC, a region important for mood and anxiety regulation. iTBS was used either as a stand-alone intervention or in combination with other forms of rTMS, such as high-frequency rTMS (10 Hz) or additional drug treatment.

Randomized and controlled trials reported reductions in depressive symptom severity when iTBS or rTMS was administered, either alone or as an adjunct to ongoing pharmacotherapy. Most studies did not include an active comparator; control conditions, when present, included active comparators such as 10 Hz rTMS or medication-only groups. However, stimulation parameters and outcome measures varied considerably across studies, and effect sizes ranged from small to moderate when reported. Studies comparing different stimulation protocols or active conditions suggested symptom improvement in both depressive and anxiety domains.

It should be noted that the absence of sham control limits causal inference and increases susceptibility to nonspecific treatment effects. Where reported, standardized effect sizes for depressive symptom reduction ranged from small to moderate; nevertheless, several studies did not provide effect size estimates, limiting cross-study comparison.

Stimulation parameters varied across studies. Most studies used between 600 and 1200 pulses per session, using the standard theta-burst pattern (50 Hz bursts repeated at 5 Hz). Stimulation intensity ranged from 80% to 120% of resting motor threshold (RMT), adjusted according to protocol and participant tolerance. Treatment duration was 2 to 6 weeks, five sessions per week, for a total of 10 to 30 sessions. Control conditions included, comparison with 10 Hz rTMS and medication-only groups.

Clinical outcomes were assessed using the following rating scales: HAM-D, HAM-A, HADS, PHQ-9, and GAD-7. Response was defined as a ≥50% reduction in symptom severity, while remission criteria typically included HAM-D ≤ 7 or GAD-7 < 5.

Response rates varied from 38% to nearly 60%, while remission rates were generally lower, ranging from <10% to 43%. Comparisons of iTBS and rTMS at 10 Hz have yielded inconsistent results, with some studies reporting equivalent clinical benefits and others suggesting superior outcomes with high-frequency stimulation.

An open-label study by Zhang et al. [13] reported only outcomes based on paired *t*-tests, with results on HAD-A scores decreased from a mean of 8.78 (SD = 4.09) at baseline to 6.16 (SD = 3.64) following the iTBS sessions. Depression scores decreased on average from 10.76 (SD = 3.90) before procedures to 7.75 (SD = 3.70) after the iTBS sessions. Response and remission rates were not reported, which limits the ability to compare iTBS effectiveness with other interventions.

Several factors have been noted to influence treatment outcomes. Age and lower baseline symptom severity were associated with better response in some studies. The impact of comorbid anxiety was mixed, with some evidence suggesting a reduced response, while other studies reported substantial improvement in anxiety symptoms after iTBS. Concomitant use of medication played a modulating role, with patients receiving bupropion showing higher rates of response and remission, whereas antipsychotics, anticonvulsants, and benzodiazepines were associated with diminished clinical improvement. These findings indicate that the pharmacological context may be an important moderator of the efficacy of iTBS.

### 4.6. Primary Clinical Outcomes: Changes in Depressive and Anxiety Symptoms

iTBS produced improvements in depressive and anxiety symptoms in all included studies. Reported response rates (≥50% reduction in symptoms) ranged from approximately 38% to 58%, while remission rates varied more widely, from less than 10% to more than 40%, depending on study conditions.

In all studies, anxiety symptoms improved along with depressive symptoms, and in several cases, measures of anxiety had greater reductions. Studies that incorporated iTBS into combination therapy settings reported greater symptom improvement compared with those that used iTBS as a monotherapy.

Only a minority of included studies reported mechanistic or biological measures, such as neurophysiological or imaging-based outcomes. These measures were heterogeneous, inconsistently reported, and insufficient for comparative synthesis. Consequently, no integrative conclusions regarding the mechanistic effects of rTMS could be drawn from the available data.

Comparisons between iTBS and high-frequency rTMS were mixed, with some studies showing comparable or superior symptom reductions with iTBS, while others found no significant difference between stimulation modalities. Despite variability in results, all studies reported clinically significant reductions in at least one major symptom domain. In conclusion, iTBS is associated with significant reductions in the severity of depression and anxiety, although the magnitude of improvement varies across studies.

## 5. Discussion

This systematic review pooled evidence from studies evaluating iTBS in adults with MDD presenting with comorbid anxiety disorders or clinically significant anxiety symptoms.

Across all included studies, iTBS was consistently associated with clinically meaningful reductions in both depressive and anxiety symptom severity. Across the included studies, iTBS was consistently associated with clinically meaningful reductions in both depressive and anxiety symptom severity. Reported response rates generally ranged from approximately 38% to 58%, while remission rates varied more widely, from about 10% to 43% [13,15,17,23]. Collectively, these findings suggest that iTBS represents a feasible, well-tolerated, and time-efficient neuromodulation approach, offering a practical alternative to conventional high-frequency (10 Hz) rTMS, particularly in clinical settings where treatment burden and session duration are important considerations. Differences in efficacy across studies appear to reflect variations in stimulation parameters, treatment duration, sample characteristics, and concomitant medication use.

Studies that used higher stimulation intensities, higher pulse counts, or multimodal protocols—such as iTBS combined with low-frequency rTMS or antidepressants—tended to report stronger clinical outcomes [17,22]. In contrast, shorter or lower-intensity stimulation paradigms produced more modest improvements [13], highlighting the importance of appropriate dosing. Several studies also indicated that younger age, lower initial severity, and bupropion use predicted better outcomes, while benzodiazepines, antipsychotics, and anticonvulsants were associated with a diminished response [17], highlighting the modulating influence of the pharmacological context. Multimodal approaches such as pharmacotherapy and neuromodulation, together predict a clinically meaningful improvement in anxiety symptoms, frequently slightly more modest than depressive symptoms alone [24,25,26].

Recent neurobiological evidence offers possible explanations for the variability in response to iTBS observed in comorbid depression and anxiety. iTBS targets DLPFC, an area essential for emotion regulation and cognitive function, which is thought to enhance control over overactive limbic regions such as the amygdala [21,27].

However, individuals with high or chronic anxiety often exhibit elevated amygdala activation, reduced prefrontal inhibitory capacity, patterns associated with persistent threat monitoring, and impaired cognitive control [2,4].

Such dysregulation of neural activity may limit the ability of iTBS to produce lasting plastic changes, reducing its therapeutic efficacy. Individuals with higher baseline anxiety traits are less likely to achieve a favorable response, and, in particular, the association with somatic anxiety predicts poorer remission on antidepressants unless early signs of improvement are observed [28]. Furthermore, anxiety-related hyperactivation may impair long-term potentiation (LTP) mechanisms that underlie the excitatory effects of iTBS, thereby diminishing the neurophysiological response to stimulation.

These mechanistic considerations align with clinical findings that comorbid anxiety may attenuate antidepressant response and may help explain variability in symptom improvement across individuals. In contrast, when prefrontal-limbic connectivity is successfully modulated, concomitant improvements in depressive and anxiety symptoms are often observed, suggesting that the integrity of the underlying network may serve as a critical determinant of treatment outcome. The evidence shows that these networks present aberrant coupling between the Default Mode Network (DMN), limbic, and frontoparietal regions, and that both baseline connectivity patterns and their subsequent normalization are correlated with clinical improvement [29,30]. Notably, TMS and electroconvulsive therapy (ECT) normalize subgenual cingulate–DMN hyperconnectivity and restore DMN–frontoparietal coupling, with the extent of these changes correlating with symptom improvement [31,32]. Thus, DMN circuits, together with the limbic and frontoparietal circuits, represent distinctive neurobiological features of depressive–anxiety states and can predict the treatment response. Brain network analyses indicate that even minor variations in stimulation targets may modulate distinct neural pathways related to anxiety and depression, thus having a significant influence on clinical outcomes. The “Beam F3” target is more reliable in TMS for depression [33], whereas targeting brain regions using the “5.5 cm method” could alleviate trait anxiety [34].

Comparative evidence between iTBS and 10 Hz rTMS has been mixed: some studies reported similar or even better improvements with iTBS [13,15], while others found no significant difference after adjusting for clinical predictors [23]. These inconsistent results are likely due to differences in protocols, sample diversity, and variable diagnostic profiles, highlighting the need for standardized stimulation parameters and stratified study designs.

The findings indicate that iTBS is an effective and efficacious intervention for depression with comorbid anxiety, especially within multimodal treatment approaches, and may exert its therapeutic effects by modulating prefrontal-limbic circuits [21], although further neurobiological research is needed to confirm this mechanism.

Future research should focus on randomized, pre-registered clinical trials with standardized stimulation protocols to facilitate comparability. The use of multimodal measures, including neuroimaging and biomarkers, will help to understand the mechanisms of treatment response. Long-term assessments are needed to establish the durability of effects and relapse prevention. In addition, individualized stimulation, tailored to dosing parameters, targets, and clinical factors such as comorbid anxiety or medication, may increase the accuracy and efficacy of iTBS.

## 6. Limitations of the Study

Although this review followed PRISMA guidelines and a preregistered protocol, the applied search strategy prioritized studies explicitly addressing depression–anxiety comorbidity or systematically reporting anxiety outcomes. As a result, many iTBS trials focused primarily on depression—where anxiety was assessed only as a secondary outcome without prominent indexing—may not have been captured. The relatively small number of included studies, therefore, reflects both a true limitation of the current evidence base for formally defined comorbidity and a trade-off inherent in maintaining conceptual specificity. Future systematic reviews may benefit from broader search strategies followed by post hoc stratification based on anxiety outcome reporting.

The number of studies specifically investigating iTBS in individuals with comorbid MDD and anxiety disorders is quite small. This limits statistical power and reduces the generalizability of the results. Many studies primarily target depressive symptoms and report anxiety outcomes only as secondary measures [13,17,23]. An important limitation of the current evidence base is the heterogeneity in both study design and the operationalization of anxiety. While most studies assessed anxiety dimensionally using symptom severity scales within MDD cohorts, fewer studies examined formally diagnosed comorbid anxiety disorders. Consequently, the findings may be more representative of MDD with prominent anxiety symptoms rather than categorical comorbid anxiety disorders.

There is substantial variation across studies in stimulation parameters, number of pulses, session intensity and frequency, as well as treatment duration and stimulation targets [15,22].

Inconsistencies in methodological rigor have been identified. Several studies did not report detailed randomization procedures, implemented partial or absent blinding, or provided incomplete information on the use of concomitant medication, all factors that may compromise internal validity [17,21]. Small sample sizes in some studies further increased the likelihood of type II errors and limited the detection of subtle but clinically significant effects [13,15].

Another limitation is the lack of long-term follow-up assessments. Most studies assessed outcomes only during or immediately after treatment, making it difficult to determine the durability of clinical improvements or the likelihood of relapse after iTBS [23].

The lack of standardized measures and limited inclusion of neurobiological assessments reduce the relevance and interpretability of the results. The small number of included studies and the marked heterogeneity in stimulation parameters, study designs, outcome measures, and concomitant treatments substantially limit generalizability and preclude quantitative synthesis. Future research should use standardized protocols, larger samples, mechanistic outcome measures, and long-term follow-up to strengthen the evidence base and clarify the role of iTBS in the treatment of depression–anxiety comorbidity.

## 7. Conclusions

This review summarizes the current evidence supporting the idea that iTBS is a promising neuromodulatory intervention for people with comorbid MDD and anxiety disorders.

Taken together, the available evidence suggests that rTMS may be associated with improvements in depressive symptoms in individuals with MDD and elevated anxiety symptoms, particularly when used as an adjunctive intervention. However, conclusions regarding efficacy remain preliminary due to the small number of studies, methodological heterogeneity, and variability in outcome reporting. iTBS has demonstrated promising therapeutic potential, with several studies reporting significant reductions in both depressive and anxiety symptoms. However, results remain variable, largely due to differences in study design, stimulation parameters, and participant characteristics.

Although iTBS appears to provide comparable antidepressant efficacy to rTMS, the substantially shorter administration time may confer a practical advantage and may also improve treatment efficiency and patient adherence. Evidence suggests that approaches combining iTBS with low-frequency rTMS or pharmacotherapy may improve treatment outcomes in certain subgroups, although further rigorous evaluation is needed.

Clinically, the results suggest the utility of iTBS for addressing the complex symptom profiles characteristic of comorbid depression and anxiety, which are often associated with treatment resistance and dysfunction of overlapping neural circuits. The lack of standardization of protocols, heterogeneity of stimulation targets, and limited long-term follow-up data underscore the need for further investigation.

Future research should prioritize large, preregistered RCTs employing standardized stimulation parameters, incorporate neuroimaging and biomarker-based assessments to elucidate mechanisms of action, and identify predictors of treatment response to support personalized neuromodulation strategies. As the evidence base expands, systematic evaluations such as this review will play a key role in refining clinical protocols and advancing iTBS toward broader implementation as an evidence-based treatment for comorbid mood–anxiety disorders.

## Figures and Tables

**Table 1:** Inclusion criteria.

Domain	Eligibility Criteria
Study Design	Full-text, peer-reviewed randomized controlled trials (RCTs), clinical trials, or controlled clinical studies.
Population	Adults (≥18 years) diagnosed with MDD, anxiety disorders, or comorbid MDD–anxiety according to DSM or ICD criteria.
Intervention	iTBS or related TBS protocols. cTBS was included when mechanistically relevant to iTBS outcomes.
Language and Accessibility	Articles published in English and available in full text.

**Table 2:** Exclusion criteria.

Domain	Eligibility Criteria
Study Design	Studies involving non-human participants or in vitro experimental models
Population	Studies including pediatric populations
Intervention	Trials using only standard rTMS without any iTBS or TBS component
Language and Accessibility	Case reports, editorials, commentaries, conference abstracts, or protocol-only manuscripts

**Table 3:** GRADE Summary of Findings for the Efficacy and Safety of iTBS in Major Depressive Disorder with Anxiety Symptoms.

Outcome	No. of Studies (Design)	No. of Participants	Risk of Bias	Inconsistency	Indirectness	Imprecision	Comments	Certainty of Evidence (GRADE)
Reduction in depressive symptoms (HDRS/MADRS/PHQ-9 scores)	6 (one RCT; one controlled trial; 3 open-label studies; one retrospective study)	Li et al. [21] = 159. Zhang et al. [13] = 37. Sun et al. [22] = 75; Aoun et al. [18] = 196; Trevizol et al. [23] = 388. Lee et al. [15] = 42	Serious (RoB2: lack of blinding; inclusion of open-label and retrospective studies)	Serious (heterogeneous iTBS parameters and protocols)	Not serious	Serious (small samples, wide confidence intervals)	Favorable effects reported, but evidence remains inconclusive	Very low
Reduction in anxiety symptoms (HAM-A, BAI)	6 (one RCT; one controlled trial; 3 open-label studies; one retrospective study)	Li et al. [21] = 159. Zhang et al. [13] = 37. Sun et al. [22] = 75. Aoun et al. [17] = 196; Trevizol et al. [24] = 388. Lee et al. [15] = 42	Serious	Serious	Serious (anxiety defined as symptom severity rather than formal diagnosis)	Serious	Most studies did not include formally diagnosed anxiety disorders	Very low
Response rate	4 (mixed designs)	Li et al. [21] = 159; Aoun et al. [18] = 169. Trevizol et al. [23] = 388. Lee et al. [15] = 42	Serious	Serious	Not serious	Serious	Response criteria varied substantially across studies	Very low
Remission rate	4 (mixed designs)	Li et al. [21] = 159. Aoun et al. [17] = 169. Trevizol et al. [23] = 388. Lee et al. [15] = 42	Serious	Serious	Not serious	Very serious (few events, unstable estimates)	Limited number of remission events	Very low

**Table 4:** Summary of study characteristics, stimulation parameters, target sites, and clinical outcomes across included trials.

Study (Year)	Protocol/Intervention	Stimulation Parameters	Target Area	Population	Response	Remission	*p* Value
Li et al. [21]	2–4 weeks iTBS add-on treatment	1200 pulses, 50 Hz, 80–120% MT	Left DLPFC	159 adults (18–65 y), MDD (first episode and recurrent); add-on iTBS + antidepressants + psychotherapy	HDRS response 57.86% HAM-A response 65%	HDRS remission 28.93%, HAM-A remission 59.15%	*p* > 0.05
Zhang et al. [13]	10 iTBS sessions	30% MT, 12,000 pulses	Left DLPFC	37 adults (18–65 y) with anxiety and depression	Not reported	Not reported	*p* < 0.001
Sun et al. [22]	Combined therapy (iTBS + 1 Hz rTMS + sertraline (50–100 mg); 5 sessions/week × 3 weeks (15 total sessions)	Each session 1600 pulses (800 per site); 90% resting motor threshold	Left DLPFC (iTBS), 1 hz right DLPFC	75 adults with depression or anxiety-related disorders;	Not reported	Not reported	*p* < 0.001
Aoun et al. [17]	30 sessions over 3 weeks; 1–2 sessions/day; intervals: HF/LF 15–20 min, iTBS 20–40 min HAM-D, SDS, BAI at baseline, after 10, 20, and 30 sessions.	HF: 10 Hz L-DLPFC, 120% RMT LF: 1 Hz R-DLPFC, 120% RMT iTBS: 80% RMT L	DLPFC	196 adults with MDD rTMS L/R DLPFC (Brodmann areas 9 and 46) via neuronavigation	HF-62.3% LF-71.4% iTBS-49.5% Overall-57.7%	Overall 42.3%; HF-50.9%; LF-42.9%, iTBS-37.6%; meds influence (bupropion ↑, antipsychotics/anticonvulsants ↓)	*p* < 0.05
Trevizol et al. [23]	5 sessions/week, 20 sessions over 4 weeks; up to 10 additional sessions over 2 weeks for participants with ≥30% HAM-D improvement but not remission. HAM-D; BSI-A at baseline	10 Hz rTMS, DLPFC, 120% RMT iTBS, 120% RMT 600 pulses/session	Left DLPFC	Adults (18–65 y) with MDD; *n* = 388 (iTBS: 199, 10 Hz rTMS: 189), history of 1–3 failed antidepressant trials or intolerance to ≥2 trials.	rTMS with no comorbid anxiety disorder (54.1%); rTMS with comorbid panic disorder (35%) or generalised anxiety disorder (47.3%)	28.6% (clinical and demographic factors (employment status, number of prior medications, baseline depression and anxiety severity) are stronger predictors of remission probability)	*p* < 0.05
Lee et al. [15]	30 sessions (5/week), plus 6 taper sessions (2–3/week), no sham group; Assessment at baseline, every ~2 weeks during treatment, and end of acute treatment series	iTBS, 120% RMT, 600 pulses per session	Left DLPFC	*n* = 42 41.7 years mean age 59.5% female	PHQ-9: ≥50% reduction 38.1% GAD-7: ≥50% reduction 33.3% Patient-reported outcome: “Meaningful improvement” (yes/no) Patient-reported meaningful improvement (PRO): 57.1%	(PHQ-9 < 5): 9.5% (GAD-7 < 5): 28.6%	*p* < 0.001

Note: The upward arrow (↑) reflects a relative increase or positive association, whereas the downward arrow (↓) reflects a relative decrease or negative association in relation to the reported outcome.

**Table 5:** Risk of Bias Assessment and Methodological Quality of Included Studies.

Study (Year)	Design	Randomization	Blinding (Participants/Assessors)	Attrition Bias	Selective Reporting	Sample Size Adequacy	Overall Quality Rating
Sun et al. [22]	Controlled trial (iTBS + 1 Hz rTMS + sertraline)	Unclear	No/Yes	Moderate	Unclear	Moderate (*n* = 75)	Moderate
Trevizol et al. [23]	RCT (iTBS vs. 10 Hz rTMS)	Adequate	Yes/Yes	Low	Low	Large (*n* = 414)	High

**Table 6:** Risk of Bias Assessment of open-label and retrospective studies (ROBINS-I).

Study (Year)	Confounding	Selection of Participants	Classification of Intervention	Deviations from Intended Intervention	Missing Data	Measurement of Outcomes	Selection of Reported Outcomes	Selection of Reported Results	Overall ROB
Li et al. [21]	Serious	Moderate	Low	Moderate	Moderate	Moderate	Serious	Moderate	Serious
Zhang et al. [13]	Serious	Moderate	Low-Moderate	Moderate	Moderate	Moderate	Moderate	Moderate	Serious
Lee et al. [15]	Moderate	Moderate	Low	Moderate	Low-Moderate	Moderate	Moderate	Moderate	Moderate
Aoun et al. [17]	Moderate	Moderate	Moderate	Low	Moderate	Serious	Moderate	Moderate	Moderate

**Table 7:** Summary of participant demographics, diagnostic profiles, study designs, and iTBS intervention protocols across the included studies.

Study (Year)	Sample Size (N)	Mean Age (Years)	Diagnosis/Comorbidity	Study Design	Intervention Protocol	Treatment Duration	Stimulation Intensity	Comparison/Control
Li et al. [21]	159	18–65	MDD (first episode and recurrent), comorbid anxiety	Open label	Add-on iTBS (left DLPFC) + antidepressants + psychotherapy	2–4 weeks	80–120% MT, 1200 pulses	No sham
Zhang et al. [13]	37	18–65	Anxiety and depression	Open label	10 sessions of iTBS (left DLPFC)	2 weeks	30% MT, 12,000 pulses total	No sham
Sun et al. [22]	75	18–60	Depression or anxiety-related disorders	Controlled trial	Combined therapy (iTBS + 1 Hz rTMS + sertraline 50–100 mg)	3 weeks (15 sessions)	90% RMT, 1600 pulses/session	Conventional therapy (sertraline only)
Aoun et al. [17]	196	18–65	MDD	Retrospective	30 sessions (HF/LF/iTBS protocols) over 3 weeks	3 weeks	80–120% RMT	No sham
Trevizol et al. [23]	388	18–65	MDD (treatment-resistant)	Multicenter RCT	iTBS vs. 10 Hz rTMS (left DLPFC)	4–6 weeks	120% RMT, 600 pulses	10 Hz rTMS (active comparator)
Lee et al. [15]	42	41.7 ± 10.2	MDD with comorbid GAD-7	Open label	30 sessions + 6 taper iTBS (left DLPFC)	6 weeks	120% RMT, 600 pulses/session	No sham

## Data Availability

No new data were created or analyzed in this study.

## Supplementary Materials

The following supporting information can be downloaded at https://www.mdpi.com/article/10.3390/brainsci16020167/s1, Table S1: PRISMA checklist; Table S2: Electronic search strategy.

## Abbreviations

The following abbreviations are used in this manuscript:

BAI	Beck Anxiety Inventory
BDI	Beck Depression Inventory
BSI-A	T Brief Symptom Inventory–Anxiety subscale
cTBS	Continuous Theta Burst Stimulation
DLPFC	Dorsolateral Prefrontal Cortex
DMN	Default Mode Network
DSM	Diagnostic and Statistical Manual of Mental Disorders
ECT	Electroconvulsive Therapy
fMRI	Functional Magnetic Resonance Imaging
fNIRS	Functional Near-Infrared Spectroscopy
GAD-7	Generalized Anxiety Disorder 7-item scale
GRADE	Grading of Recommendations Assessment, Development and Evaluation
HAM-A	Hamilton Anxiety Rating Scale
HAM-D/HDRS	Hamilton Depression Rating Scale
HADS	Hospital Anxiety and Depression Scale
HF-rTMS	High-Frequency Repetitive Transcranial Magnetic Stimulation
ICD	International Classification of Diseases
iTBS	Intermittent Theta Burst Stimulation
LF-rTMS	Low-Frequency Repetitive Transcranial Magnetic Stimulation
LTP	Long-Term Potentiation
MDD	Major Depressive Disorder
MT/RMT	(Resting) Motor Threshold
PHQ-9	Patient Health Questionnaire 9-item scale
PRISMA	Preferred Reporting Items for Systematic Reviews and Meta-Analyses
PRO	Patient-Reported Outcome
PROSPERO	International Prospective Register of Systematic Reviews
RCT	Randomized Controlled Trial
rTMS	Repetitive Transcranial Magnetic Stimulation
RoB 2	Risk of Bias tool, version 2
SDS	Symptom Distress Scale
SSRIs	Selective Serotonin Reuptake Inhibitors
TBS	Theta Burst Stimulation
